# The American Proctologic Society

**Published:** 1902-07

**Authors:** 


### *SOCIETY REPORTS.*

---

### THE AMERICAN PROCTOLOGIC SOCIETY.

The fourth annual meeting of the American Proctologic Society was called to order in the parlors of the U. S. Hotel, Saratoga Springs, N. Y., by the President, Dr. Thomas Charles Martin of Cleveland, Ohio, at 2 P.M., Tuesday, June 10, 1902.

Dr. William Bodenhamer, of New York, and Dr. Edmund Andrews, of Chicago, were elected honorary members of the Society.

The following were elected active members of the Society: Dr. A. Teirlinck, Ghent, Belgium; Dr. William L. Dickinson, Saginaw, Mich.; Dr. J. M. Frankenberger, Kansas City, Mo.; Dr. John T. Jelks, Memphis, Tenn.

Drs. Mathews and Beach were elected a Committee on Publication of Transactions.

The following officers were elected to serve the ensuing year:

President—Dr. Samuel T. Earle, Baltimore, Md.

Vice-President—Dr. Floyd W. McRae, Atlanta, Ga.

Secretary and Treasurer—Dr. William M. Beach, Pittsburg.

Executive Council—Dr. George J. Cook, Chairman, Indianapolis, Ind.; Dr. Lewis H. Adler, Philadelphia, Pa.; Dr. Thomas Charles Martin, Cleveland, Ohio.

Adjourned to meet at the time and place of the American Medical Association.

President's Address: "The Relation of the Rectal Valve to Obstipation. A Clinical Research." Dr. Charles Martin of Cleveland.

A glance at this table discovers that of the forty cases operated upon prior to two years ago, two were not improved, one of these had no complications discoverable by me, the other was subject to recurrent invagination of the sigmoid to the rectum; five patients were improved, three of these had complications, one of these had gonorrheal peritonitis and malignant disease of the cervix uteri.

Another has been subjected twice to laparotomy and had Meckel's diverticulum removed and oophorectomy performed, and the third had movable right kidney and an ovarian tumor; of the six patients who suffered recurrence of obstipation, three were those whose condition after operation had been improved only; of the remaining three recurrences, one had been subjected to hysterectomy, and the other two (cases 15 and 26) I have not been able to report. In a total of forty-five cases, five were improved and thirty-three cured.

"Dietary in the Treatment of Rectal Diseases." By A. P. Buchman, M.D., Ft. Wayne, Ind.

Beginning with hemorrhoids and ending with proctitis, catarrhal or otherwise, the whole pathologic ensemble is primarily referable to what has been over a long time, and what is now daily ingested in the food supply. Indigestion, even in its most elementary form, is overlooked and underestimated. Digestion is not fermentation. It is a process of decomposition of molecular particles and a recomposition of chemical elements by which toxins and toxic substances are formed. Carbon dioxide in excess in living tissue for too long a time will produce paralysis as to function and finally organic disintegration. Catarrhal proctitis is often many feet away from the point treated. Give well regulated diet for chronic constipation. Internal hemorrhoids disappear under palliative treatment and regulated diet. Case. Thirty per cent. protargol to mucous membrane. Daily colon bath. Open air. Rest. Morbid conditions that are attributable to an irregular and imperfect diet. Regarding the intestinal tract as a single organ. Difference between digestion and fermentation. By scientific dietary regulation certain surgical procedures in this field can be reduced to the smallest common multiple.

Cases reported by writer.

Conclusion: If the dietary care of patients was prescribed with the same accuracy and care that is carried out along drug and surgical lines, the value of the method would not be the subject of as much neglect as it is at the present time.

DISCUSSION ON DR. BUCHMAN'S PAPER.

Dr. Samuel T. Earle, of Baltimore, called attention to the fact that we should reflect in these cases before resorting to the operation.

Dr. James P. Tuttle of New York: This paper justifies the position taken by Dr. Mathews and myself with regard to taking of others into the society. It is the work of these men along lines other than operative ones.

Dr. A. P. Buchman, of Ft. Wayne, closes: I heard a lecture yesterday on the stomach at the postgraduate in New York, in which the lecturer detailed the pathologic condition and then detailed the drug treatment. Each member of the class took down carefully all he had said; then he said, "the most important thing is the diet," and the books closed at once. He should have given the diet first.

"Diagnosis and Treatment of Proctitis." Howard A. Kelley of Baltimore.

The special importance of the subject alluded to lies in the fact that the affection is for the most part unrecognized and so allowed to run a chronic course. The local symptoms are often vague, and in women they give rise to a disease of the uterus or ovaries. Some of its symptoms are decidedly serious in character, such as the poisonous character of the mucous secretion of the bowel. When associated with other pelvic diseases, it is apt to be overlooked. Cause is generally obscure. The better recognition of these affections hinges on the diagnosis. If such a case is examined per vagina, empty rectum feels like a flattened fibrillated cord, easily rolled from side to side. Detail of pathology given. Topical and dietary and drug treatment and massage recommended.

"Some Unusual Causes of Proctitis and Diarrhea." Jas. P. Tuttle of New York.

Excepting atrophic catarrh almost all forms of proctitis are associated with diarrhea. Most types occur in tropical regions and are due to specific micro-organisms. Treatment, prolonged rest in bed and irrigations with fluid extract of krameria in irrigation solutions. Diet of milk and meat exclusively was followed by improvement.

"Trachymonas Intestinalis." Parasitis disappeared under irrigation of pure lime-water.

Anchylostoma intestinalis. Case. No medication followed.

Dr. S. T. Earle of Baltimore: In defense of the ground taken by bacteriologists that the role played by the ameba is the dysentery as we find it in temporate climates. Whether or not other organisms pave the way for the ameba or whether they do it themselves is a question.

Dr. George J. Cook of Indianapolis: In 1876 Andrews, of Chicago, gave statistics which were obtained directly from advertising specialists, showing that in 3,200 cases of hemorrhoids treated by injection of caustics, there were thirteen deaths due directly to treatment, besides eight cases of embolism of the liver, ten cases of dangerous hemorrhage, numerous cases of severe pain, abscess, fistule and ulceration. Since that time there has been no evidence to indicate that better results have been obtained. A report of six cases of deaths due directly to this treatment is given, covering the past eighteen months. All were treated by advertisers but subsequently came under the care of regular physicians from whom reports were received. In every case an abscess formed as the result of the injection, and septic infection followed, resulting in death in from six to ten days. In one case was pneumonia, in another thrombus of the hepatic vessels. Many of the bad results following this treatment are attributed to the methods of injection. Having the piles protruding through the anus and then injected when the vessels are distended with blood, is bad practice, because when the tumors are replaced much of the coagulated blood is forced from the pile tissue into the surrounding vessels and may cause abscess or sloughing. The better method is to use a tube speculum, and after the blood is pressed out of the tumor inject the caustic, thus avoiding the formation of blood-clots in the vessel. Many cases were treated in this way with only two unpleasant results. In these cases there was secondary hemorrhage which was easily controlled. This method of treatment is advised only in cases where a general anesthetic cannot be administered. The treatment of piles by injection is the most dangerous method in use, as is shown by statistics. The relief in the majority of cases is temporary, averaging about four years.

DISCUSSION.

Dr. J. M. Mathews of Louisville: The proctologists should either indorse this treatment or denounce it. In my infirmary to-day are two ladies, one of whom was treated for three weeks in New York by the injection plan. She does not seem to have any hemorrhoids. On examination I found a hole in the gut. Another lady had the same injection plan and the same result. I have seen two cases of violent hemorrhage, endangering the lives of the patient. I have also seen much injury done and do not believe in irrational treatment, as it is so easy to establish a dangerous condition and is not so radical a cure and indorses quack treatment. We can very well see that by injecting a tumor of this kind an artery may be injected and a thrombus established. As the author says, he would only use it where it was dangerous to give an anesthetic; I often think cases are chimerical. We have given ether or chloroform where we have had heart murmurs.

Dr. Geo. B. Evans of Dayton, O.: In eleven years he has never injected a single pile, because it is not at all radical. The doctor was influenced to take this stand by Kelcey, Tuttle and Mathews in 1891. Fully fifty per cent of the cases have had previously the carbolic acid treatment. These cases had some kind of fistula or fissure, and the doctor prefers keeping his hands off the hypodermic.

Dr. L. H. Adler, of Philadelphia, is in favor of the treatment. Some patients absolutely refuse to have any operation performed and think the profession entirely wrong in condemning the method *in toto.*

Dr. J. Rawson Pennington, of Chicago, says in his estimation there are too many "I thinks" and "I guesses" in medicine. A quack discovers or originates some method and the profession continues to condemn and condemn, and then takes it up. He has had little experience with the carbolic treatment, but on general principles does not like it. Others may have had good results with it, but the clamp and cautery and the ligature are what he gets the best results with.

Dr. Geo. J. Cook, in closing the discussion, said he used the injection method as a last resort. When he does it, he injects only one pile at a time.

The meeting then adjourned until Wednesday, June 11, at 2 P.M.

SECOND SESSION, WEDNESDAY, JUNE 11, 2 P.M.

"On the Causes and Treatment of Rectal Abscess." Wm. M. Beach of Pittsburg.

The location of an abscess is usually definite and circumscribed about the rectum by the limits of the pelvic fascia and integument. There are the marginal and deep-seated varieties. The ischio-rectal is one of the latter. Trauma, mechanical, syphilis, tubercular, chemical, thermic, secondary processes, bacteriologic, such as gonorrhea, bacillus colon communis, zymotic diseases and idiopathic, seem to be the exciting causes, which vary according to the location of the pus areas. As soon as pus is determined, freely evacuate it. Introduce intestinal antiseptic and apply cold. Never poultice.

Dr. James P. Tuttle of New York: The prompt and radical treatment of this inflammatory condition around the rectum is perhaps the means of preventing rectal diseases. Four out of five fistulas can be avoided if the peri-rectal inflammatory condition is treated in the beginning.

"The Treatment of Rectal Fistulæ by Complete Excision and Closure with Buried Catgut Sutures." Dr. Floyd W. McRae of Atlanta.

Some time ago the author advised treating fistulæ by excision of the entire fistulous tract, bringing the raw surfaces together by sutures with a view to securing healing by first intention. Many cases and authorities are cited. This method of procedure saves the victims of this disease an enormous amount of suffering. Has had very good success. Operation described and two cases reported.

DISCUSSION.

Dr. J. Rawson Pennington, of Chicago, described a modification of this operation.

Dr. S. T. Earle, of Baltimore, thinks Dr. Pennington's idea is very much more difficult than that of the author. When a fistulous tract is laid open it is not difficult to find the openings of other tracts.

Dr. L. H. Adler, of Philadelphia, congratulates Dr. McRae on his paper and Dr. Pennington on his suggestion. The doctor has had very unfortunate results with the use of this method, but he may be led to take it up later on.

Dr. George J. Cook, of Indianapolis, said that Dr. Morris, of New York, always filled the fistulous tracts with plaster of Paris and then dissected the whole thing out.

The president, Dr. Martin of Cleveland: Do not expect patients or select patients for this operation who are stout. Patients who have a thick pelvic floor are not good patients for this operation. Where there is much induration the doctor advises taking baths for some time so that the induration will be lessened. Traction is made on the fistulous tract by means of a forceps, and over the ischiorectal space a broad adhesive band of T bandage is placed.

Dr. McRae, in closing the discussion, said that he thought Dr. Pennington's method a great deal more difficult. Where there is more or less retention of pus it is unfavorable for treatment. The real points of merit in this method are the laying open of these tracts and cleaning each one separately and a thorough dissection of all the indurated tissue.

Dr. L. H. Adler, of Philadelphia, here moved that the Treasurer be authorized to pay the bill of the stenographer. Seconded and carried.

Dr. J. Rawson Pennington, of Chicago, presented pathological specimens showing the various valves of the rectum.

"Excision of Cancer of the Rectum, with Report of Two Cases." By L. H. Adler of Philadelphia.

The doctor called attention to the importance of this subject. The disease in the rectum has no marked manifestations until the trouble has progressed to such an extent that complete ablation of the growth is impossible. The author antagonizes any and all attempted excisions of the rectum for malignant disease. To assume such an attitude would be the height of folly; it would consign the patients to certain death. The author has had some two hundred and seventeen cases, and of this number has excised the rectum in three, curetted the growth in seven, and colostomized the growth in twenty-seven. Relative to the three cases of excision, one patient died within the year following operation, of cancer of the liver; another died five years after the excision at the age of seventy-seven, and at the autopsy no trace of the disease was discoverable—death being due to senility; the third patient is still alive and active—though past seventy years of age—the operation was performed six years ago. Cancer of the rectum at the stage usually discovered by the surgeon, is less amenable to the knife than cancer in other portions of the body. Operation is followed by cure in a small proportion of the cases, and the dangers following excision are great, and the results as to comfort anything but satisfactory; yet in cases seen early much good can be accomplished by the operation.

"Colotomy; Tumors Complicating It." By B. Merrill Ricketts of Cincinnati.

Colotomy may be attended with many complications, due to benign or malignant neoplasms. Reports two or three complicating cases. Hernia may complicate; hydrocele, aneurism, adhesions, and cysts are frequently found by the surgeon.

DISCUSSION.

Dr. J. Rawson Pennington, of Chicago, described a case of a malignant growth of the rectum on which a colostomy was done and the X-ray later used. For a period of five months no pus has been seen. The patient had had violent pains in the legs and abdomen, which disappeared after the X-rays had been used.

The President, Dr. Martin, also reported a similar case.

Dr. Jas. P. Tuttle, of New York, and Dr. Floyd W. McRae, of Atlanta, also reported similar cases.

Dr. L. H. Adler, of Philadelphia, in closing the discussion, said that all patients gained in weight, sometimes as much as twenty-five pounds. He also called attention to the fact that cancer was very prevalent in the sooty regions of our country.

"The Significance and Treatment of Hemorrhage from the Rectum." By A. Bennett Cooke of Nashville.

"Treatment of Hemorrhoids by Enucleation." By Geo. B. Evans of Dayton.

There are two classes of internal hemorrhoids—the capillary and the venous. Capillary is in reality a rectal tumor. Case described. The author does not believe in tamponing, nor the use of the Pennington plug. In this operation, as in all others for hemorrhoids, we fail to find an ideal one. In its application, it is no doubt restricted. In cases in which the varicosities are comparatively accessible, when from any reason there is no bowel paresis and the plug can be placed securely, and securely remain for a time, thereby insuring protection from hemorrhage and likely from infection, it is no doubt a very agreeable and satisfactory operation to both patient and operator; but to be able to determine in every instance in which apparent weakness of the operation may manifest itself, it seems likely that it would be difficult. Eleven successful cases reported

DISCUSSION.

Dr. William L. Dickinson, of Saginaw, Mich., expressed surprise at the two bad results of this operation. He packs all his cases with iodoform gauze, coated with sterilized vaseline.

Dr. J. Rawson Pennington of Chicago: A hemorrhoid is an angioma, a diseased condition in the submucosa. He expressed considerable surprise at such unfortunate results with this operation. In 225 cases he had only two hemorrhages. Usually four days after operation the patients can get up.

Dr. Geo. B. Evans, in closing, said that in his case the bleeding vessel could not be found; it was behind the tampon. He likes this operation very much better than the clamp and cautery.

"A Report on Ulcerations of the Rectum." By Samuel T. Earle of Baltimore.

A complete pathologic report was carefully gone over in detail by the author.

Dr. James P. Tuttle of New York: I move the society extend to Dr. Earle a vote of thanks for his very valuable paper on this subject. Carried.

"Some Interesting Cases of Rectal Diseases." By Louis J. Krouse of Cincinnati.

The doctor presented some interesting facts of rectal diseases—one of abscess, one of ulceration, with cicatrices, and one of ischio-rectal abscess.

