# The Treatment of Hemorrhoids with the Ligature and Clamp and Cautery

**Published:** 1902-10

**Authors:** J. R. Pennington

**Affiliations:** Chicago


### CORRESPONDENCE.

### THE TREATMENT OF HEMORRHOIDS WITH THE LIGATURE AND CLAMP AND CAUTERY. A CORRECTION.

CHICAGO, September 15, 1902.

*To the Editor Journal-Record of Medicine:*

I see in the transactions of the American Proctologic Society published in THE ATLANTA JOURNAL-RECORD OF MEDICINE, July, 1902, page 233, that I am reported as saying that I get the best results with the ligature and clamp and cautery in the treatment of hemorrhoids. That report is incorrect, and in view of the fact that I have not used either of these methods for years, I do not see how such an error could have occurred. Therefore, I beg space in your journal to record in the abstract some of the reasons why I do not employ or approve either of these methods in the treatment of this malady.

1. To employ such means in the treatment of hemorrhoids is to disregard pathology, which is the basis of modern surgery and the surgical art.

2. Both methods are very painful, in violation of modern surgical principles, and I might say barbarous. Every surgeon knows that the pain following either of these operations is agonizing and extremely severe.

3. What more filthy condition could you imagine than that of a half-dozen ligatures tied to as many hemorrhoids to become contaminated with the pus and feces that must necessarily come in contact with them during defecation and the process of sloughing, which are unavoidable, to say nothing of the stench arising from this contamination and the decomposing and sloughing tumors?

4. Could you imagine a condition that would make defecation more painful than the strangulated and swollen stumps of a halfdozen hemorrhoids projecting into the rectum, which of necessity are pushed aside and dragged upon during this act?

5. Moreover, to say nothing of the disregard for pathology, and the subsequent pain, misery, suffering, filth, stench, pus, and the long time required for recovery, I maintain that it is impossible to do a radical operation on a well-defined case of internal hemorrhoids with either ligature or clamp and cautery as ordinarily employed, and not remove an excess of mucosa and submucosa, and preserve the contour and function of the anus. Respectfully,

J. R. PENNINGTON, M.D.

---

### THE DEATH OF VIRCHOW.

On September 5, at Berlin, occurred the death of Rudolph Virchow, the greatest pathologist in the world. He was born in Schievelbein, Pomerania, October 13, 1821. Virchow's career was one of the most remarkable in the annals of medicine. For more than fifty years he has stood as an authority. He early attained prominence after his graduation at the University of Berlin, being made professor of pathology at Wurzburg at the age of 28. In 1856 he was made professor of pathology at the University of Berlin. His fame rests solely upon his scientific attainments, although his energies were by no means confined to medicine. Virchow was a politician of prominence. He was an ultra-liberal, and his extreme political views caused him to lose his rectorship of the Berlin University in 1887. In 1892 he was restored to his position. Virchow founded the German Anthropological Society, also the *Archives of Pathological Anatomy and Physiology and Clinical Medicine.* Last year he celebrated his eightieth birthday, and the occasion was made one of moment by medical men all over the civilized world, physicians from all nations vying with one another to do him honor.—*Medical News.*

